# Health-Related Quality of Life in Korean Adults with Hearing Impairment: The Korea National Health and Nutrition Examination Survey 2010 to 2012

**DOI:** 10.1371/journal.pone.0163999

**Published:** 2016-10-06

**Authors:** Min Kwan Baek, Young Saing Kim, Eun Young Kim, Ae Jin Kim, Won-Jun Choi

**Affiliations:** 1 Department of Otolaryngology-Head & Neck Surgery, Gachon University Gil Medical Center, Incheon, Republic of Korea; 2 Department of Internal Medicine, Gachon University Gil Medical Center, Incheon, Republic of Korea; 3 Department of Radiology, Gachon University Gil Medical Center, Incheon, Republic of Korea; 4 Department of Occupational and Environmental Medicine, Gachon University Gil Medical Center, Incheon, Republic of Korea; IRCCS Istituto Auxologico Italiano, ITALY

## Abstract

**Background:**

As the global population ages, disabling hearing impairment (HI) have been increased rapidly. The impact of HI on health-related quality of life (HRQoL) is of great importance to aid the development of strategic plans and to guide therapeutic interventions.

**Purpose:**

To evaluate HRQoL in Korean adults with different degrees of HI using EuroQol five-dimensional (EQ-5D) and EQ-visual analogue scale (VAS), the preference-based generic measures of HRQoL.

**Methods:**

Using a representative dataset from the Korea National Health and Nutrition Examination Survey (KNHANES) from January 2010 to December 2012, EQ-5D questionnaire and EQ- VAS scores of subjects with HI were compared with those of subjects without HI. Logistic regression analysis, with adjustment for covariates, was used to evaluate the impact of HI on HRQoL scales. HI was defined according to the hearing thresholds of pure-tone averages at 0.5, 1, 2, and 3 kHz of the better hearing ear as follows; mild HI (26 to < 40 dB) and moderate to severe HI (≥ 40 dB).

**Results:**

Of the 16,449 Korean adults in KNHANES (age, 45.0 ± 0.2 years; male, 49.7%), 1757 (weighted prevalence, 7.6%) had mild HI and 890 (3.6%) had moderate to severe HI. Subjects with HI had impaired HRQoL as compared with subjects without HI (EQ-5D, 0.96 ± 0.00 vs. 0.88±0.00 vs. 0.86 ± 0.01 for control vs. mild HI vs. moderate to severe HI, *p* < 0.001; EQ-VAS, 75.10 ± 0.18 vs. 67.48 ± 0.63 vs. 66.24 ± 0.92 for control vs. mild HI vs. moderate to severe HI, *p* < 0.001). After adjusting for socio-demographic factors (age, gender, household income, education level, presence of spouse) and health-related behaviors (smoking status, alcohol intake, regular exercise), psychological stress, and the presence of comorbidities (diabetes, hypercholesterolemia, hypertension, decreased eGFR, and tinnitus), EQ-VAS remained impaired in the moderate to severe HI group (61.72±1.69) as compared with the control group (65.68 ± 1.26, *p* = 0.004), but EQ-5D impairment disappeared (0.86 ± 0.02 vs.0.88±0.01 for moderate to severe HI vs. control, *p* = 0.058).

**Conclusion:**

After adjusting for socio-demographic and psychosocial factors and comorbidities, Korean adults with moderate to severe HI rated their health statuses lower than subjects without HI.

## Introduction

Quality of life (QoL) evaluates the general well-being of individuals using a broad multidimensional concept, which includes physical, mental, material, social, and emotional well-being [1]. Health-related quality of life (HRQoL) is used to evaluate overall QoL, which has been demonstrated to affect health and usually includes self-reported measures of physical and mental health [2].

Understanding the impact of hearing loss on HRQoL is of great importance, since hearing difficulties have negative socio-emotional and physical consequences, because they make communication difficult, cause embarrassment and loneliness, and hamper social interactions [1, 3]. Since hearing impairment (HI) is a common sensory impairment in the elderly, the prevalence of moderate to severe HI, refers to disabling HI have been increased rapidly [4, 5]. However, most of them can be successfully rehabilitated using appropriate hearing aids [6–8]. HRQoL assessments are important as they identify subgroups with relatively poor perceived health and aid the development of strategic plans, guide interventions, and enable the monitoring and effectivenesses of broad community interventions that avert more serious consequences.

Although HI has been reported to impact HRQoL negatively [1, 3, 9], HRQoL should be interpreted with caution because the preferences of health states differ across cultures [10]. In a previous study that revealed an association between hearing loss and HRQoL in the Korea adult population [11], individuals with unilateral hearing loss were evaluated and focus was placed on the relationship between HRQoL and a combination of tinnitus and unilateral hearing loss, and thus, HRQoL measurements for individuals with different degrees of hearing impairment (HI) were not evaluated. In the present study, our aim was to analyze HRQoL in Korean adults with different degrees of HI using a national representative data set.

## Methods

### Study population

We used data collated during the fifth Korea National Health and Nutrition Examination Survey (KNHANES), which was conducted by the Korean Center for Disease Control and Prevention (KCDC) from January 2010 to December 2012. KNHANES is a cross-sectional, nationally representative survey conducted to determine the health and nutritional status of the civilian, non-institutionalized, Korean population.

The KNHANES is composed of a health examination, a nutrition survey, and a health questionnaire, which includes a self-assessment of HRQoL. Participants were chosen by proportional allocation-systematic sampling with multistage stratification (age, sex, and region).

Of the 19,599 adults (> 19 years old) that participated in KNHANES (2010–2012), 16,449 completed the audiometric test, the health examination, and the health questionnaire.

KNHANES was approved by the institutional review board of the KCDC, and all participants signed a written consent form. The present study and the use of KNHANES data were approved by the KCDC.

### Audiometric measurements

Pure-tone audiometric testing was conducted using an SA 203 audiometer (Entomed; Malmo; Sweden) in a soundproof booth inside a mobile unit reserved for the KNHANES survey. Otolaryngologists, who had been trained to operate the audiometer, provided instructions to participants and supervised audiometric testing. Supra-auricular headphones were used in the soundproof booth to measure air conduction thresholds. Automated testing was performed according to a modified Hughson-Westlake procedure using a single, pure tone for 1–2 seconds. The automated hearing test involving air-conducted pure-tone stimuli, and showed good test-retest reliability and validity, which were comparable to those of manual pure-tone audio testing [12]. Participants responded by pushing a button when they heard a tone and results were automatically recorded. The frequency ranges tested were 0.5, 1, 2, 3, 4, and 6 kHz. Hearing loss was defined using pure-tone averages (PTA) at frequencies of 0.5, 1, 2, and 3 kHz as follows; mild HI as a PTA of 26 to < 40 dB and moderate to severe HI as a PTA of ≥ 40 dB of the better hearing ear [5, 13].

### HRQoL measures

HRQoL was evaluated using the EuroQol five-dimensional (EQ-5D) questionnaire, which generates assessment scores across five dimensions of health, namely, mobility, self-care, usual activities, pain/discomfort, and anxiety/depression [14]. Responses in each dimension were divided into three categories; no problem, moderate problem, or extreme problem. Average scores of the EQ-5D index were calculated to assess HRQoL, which is a preference-based health status index [15–17]. HRQoL should be interpreted with caution because HRQoL has socially and culturally dependent characteristics [10]. Since the preference weights of Koreans are quite different from those of Caucasians, we used Korean specific preference weight to generate EQ-5D index scores [15]. Average scores of the EQ-5D index ranged from -0.17 to 1, where 1 indicates no problem in any of the five dimensions, zero indicates death, and negative values indicate health statuses worse than death. In addition, respondents assessed their health statuses using a Visual Analogue Scale (VAS), which ranged from 0 (worst imaginable health state) to 100 (best imaginable health state) [14].

### Independent variables

Independent variables included socio-demographic variables (age, sex, spouse status, education level, and household income) and health-related life style factors (drinking, smoking, and exercise), and psychological stress [18, 19]. Comorbidities included hypertension, diabetes mellitus, hypercholesterolemia, and decreased estimated glomerular filtration rate (eGFR) [20]. Tinnitus was also considered, because it is common in HI and can adversely affect HRQoL [11].

Unmarried, separated, widowed, and divorced subjects were allocated a ‘‘no spouse” status. Household income level was divided into national quartile groups (lowest quartile, yes/no). Education level was categorized as elementary school or lower, and middle school or higher. Drinking behavior was assessed using the Alcohol Use Disorders Identification Test (AUDIT, in which scores of ≥ 12 and < 12 indicate heavy and non-heavy drinkers, respectively) [21]. Current smokers (yes/no), routine exercise (yes/no) and level of psychological stress (moderate or extreme / no or mild) were also identified. Routine exercisers were defined as people that performed at least low-intensity physical activity, which was defined as walking or commuting for > 30 minutes more than 3 times per week.

Hypertension, diabetes, hypercholesterolemia, and decreased eGFR were defined as follows; hypertension (blood pressure ≥ 140/90 mmHg or the use of antihypertensive medication), diabetes (self-reported physician’s diagnosis, the use of hypoglycemic agents including insulin, or a fasting blood glucose of ≥ 126 mg/dL), hypercholesterolemia (fasting total cholesterol ≥ 240 mg/dL or on medication), decreased eGFR (eGFR < 60 mL/min/1.73m^2^; as defined by eGFR (mL/min/1.73m^2^) = 186.3 X (S_Cr_/88.4, *μ*mol/l)^-1.154^ X Age^-0.203^ X 0.742 (if female) [22]). Tinnitus was defined as a “yes” response to the question “Have you heard a sound (buzzing, hissing, ringing, humming, roaring, or machinery noise) originating in your ear during the past year?”

### Statistical analysis

Results are presented as percentages (± standard errors of percentages) for categorical variables and as estimated means (± standard errors of means) for continuous variables. Categorical variables and continuous variables were compared using the chi-square test or the Student’s t-test, respectively. A general linear model was used to determine adjusted mean values for subjects with different degrees of HI and for control; *p* and 95% confidence interval (CI) values were corrected using Bonferroni’s method in case of multiple testing. In addition, unadjusted and adjusted odds ratios (ORs) for poor QoL (moderate or extreme problems) respondents of the five dimensions of the EQ-5D were also evaluated. *P* values of < 0.05 (two sided) were considered significant. All analyses were adjusted for the complex survey design in KNHANES using the complex sample analysis program in PASW 18.0 (SPSS Inc., Chicago, Illinois, USA).

## Results

### General characteristics of the study population

Characteristics of the study sample are shown in Table 1. Of the 16,449 subjects (age, 45.0 ± 0.2 years; male, 49.7%), 1757 had mild HI and 890 had moderate to severe HI, which represented weighted prevalences of 7.6% and 3.6%, respectively, in the adult Korean population, and 31.9% and 19.6%, respectively, in elderly (≥ 65 years) Koreans.

**Table 1:** Socio-demographic and clinical characteristics of the study population according to the presence of hearing impairment (HI).

Characteristics	No HI (n = 13,802)	Mild HI (n = 1,757)	Moderate to severe HI (n = 890)	*p* value
**Age, years**	42.30 (0.20)	64.91 (0.43)	68.94 (0.57)	< 0.001^†^
**Elderly ≥ 65 years (%)**	7.2 (0.3)	55.6 (1.6)	70.7 (2.2)	< 0.001*
**Sex, male (%)**	49.4 (0.5)	52.0 (1.5)	51.4 (2.2)	0.172*
**Absence of spouse (%)**	9.4 (0.4)	25.5 (1.3)	32.6 (2.0)	< 0.001*
**Education ≤ Elementary school (%)**	12.8 (0.4)	54.0 (1.6)	65.3 (2.1)	< 0.001*
**Income: Lowest quartile (%)**	12.1 (0.5)	39.2 (1.5)	46.5 (2.2)	< 0.001*
**Employed: No (%)**	33.5 (0.6)	49.8 (1.6)	58.0 (2.3)	< 0.001*
**Heavy drinking (%)**	23.9 (0.5)	21.8 (1.6)	19.4 (2.2)	0.084*
**Smoking: Current smoker (%)**	59.2 (0.8)	41.8 (2.1)	42.0 (2.8)	< 0.001*
**Routine exercise: No (%)**	91.0 (0.4)	91.2 (0.9)	91.4 (1.3)	0.926*
**Hypertension (%)**	23.3 (0.5)	53.4 (1.6)	59.8 (2.1)	< 0.001*
**Diabetes (%)**	7.0 (0.3)	18.6 (1.2)	21.2 (1.8)	< 0.001*
**Hypercholesterolemia (%)**	11.5 (0.3)	20.6 (1.3)	16.2 (1.6)	< 0.001*
**eGFR < 60**	1.0 (0.1)	6.2 (0.7)	9.2 (1.1)	< 0.001*
**Tinnitus (%)**	18.9 (0.5)	36.6 (1.5)	45.1 (2.1)	< 0.001*
**Stress: moderate to severe (%)**	28.2 (0.5)	24.4 (1.3)	20.6 (1.8)	< 0.001*
**EuroQoL 5-D index**	0.96 (0.00)	0.88 (0.00)	0.86 (0.01)	< 0.001^†^
**EuroQoL visual analog scale**	75.10 (0.18)	67.48 (0.63)	66.24 (0.92)	< 0.001^†^

Values are means (standard errors of means) or percentages (standard errors of percentages).

*Pearson’s chi-squared test.

^†^Student’s t test.

Subjects with moderate to severe or mild HI were older than the 13,802 subjects without HI (control) (age, 68.9 ± 0.57 years for moderate to severe HI vs. 64.9 ± 0.4 years for mild HI vs. 42.30 ± 0.20 years for control, *p* < 0.001). Gender ratios were not significantly different between the moderate to severe HI, mild HI, and control groups (*p* = 0.172). Subjects with HI were less likely to have a spouse, more likely to have a lower level of education, and had lower household incomes (all *p* values < 0.001). Furthermore, subject with HI were more likely to be current smokers, to be exposed to moderate to severe stress, and to have comorbidities. The prevalence of heavy drinkers and those that exercised regularly were no different in the moderate to severe HI, mild HI, and control groups (*p* = 0.084 and *p* = 0.926, respectively).

### HRQoL in the moderate to severe HI, mild HI, and control

**Fig 1:**
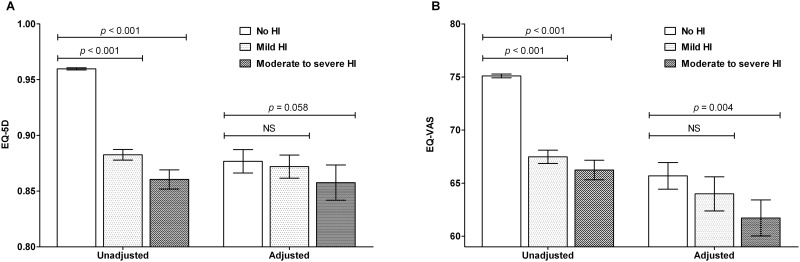
Health-related quality of life scores of subjects with mild hearing impairment (HI) and moderate to severe HI and subjects without HI. (a) EQ-5D and (b) EQ-VAS; values were adjusted for socio-demographic factors, health-related life style behaviors, psychological stress, and the presence of comorbidities. The bar graph shows means (± standard errors of means). Abbreviations; EQ-5D, EuroQol five-dimensional questionnaire; EQ-VAS, EQ-visual Analogue Scale; NS, non-significant.

HRQoLs in the HI and control groups are shown in Table 2. Overall unadjusted HRQoL was lower in the HI groups (EQ-5D, 0.96 ± 0.00 vs. 0.88 ± 0.00 vs. 0.86 ± 0.01 for control vs. mild HI vs. moderate to severe HI, *p* < 0.001; EQ-VAS, 75.10 ± 0.18 vs. 67.48 ± 0.63 vs. 66.24 ± 0.92 for control vs. mild HI vs. moderate to severe HI, *p* < 0.001) (Fig 1). After adjusting for socio-demographic factors and health-related behaviors, psychological stress, and the presence of comorbidities, EQ-VAS remained impaired in the moderate to severe HI group (61.72 ± 1.69) as compared with the control group (65.68 ± 1.26, *p* = 0.004), however, EQ-5D impairment disappeared (0.86 ± 0.02 vs. 0.88 ± 0.01 in the moderate to severe HI group vs. the control group, *p* = 0.058) (Fig 1).

**Table 2:** Health-related quality of life scales in subjects with and without hearing impairment.

	EQ-5D Index				EQ-VAS			
	No HI (n = 13,802)	Mild HI (n = 1,757)	Moderate to severe HI (n = 890)	*p* value	No HI (n = 13,802)	Mild HI (n = 1,757)	Moderate to severe HI (n = 890)	*p* value
**Unadjusted**	0.96 (0.00)	0.88 (0.00)	0.86 (0.01)	< 0.001	75.10 (0.18)	67.48 (0.63)	66.24 (0.92)	< 0.001
**Model 1**	0.95 (0.00)	0.92 (0.00)	0.91 (0.01)	< 0.001	74.75 (0.18)	70.15 (0.67)	69.48 (0.94)	< 0.001
**Model 2**	0.89 (0.01)	0.88 (0.01)	0.87 (0.02)	0.045	67.89 (1.16)	65.74 (1.53)	63.81 (1.60)	0.003
**Model 3**	0.88 (0.01)	0.87 (0.01)	0.86 (0.02)	0.041	66.23 (1.27)	64.30 (1.63)	61.76 (1.70)	0.002
**Model 4**	0.88 (0.01)	0.87 (0.01)	0.86 (0.02)	0.138	65.68 (1.26)	63.99 (1.61)	61.72 (1.69)	0.009

Values are means (standard errors of means).

Abbreviations: EQ-5D, EuroQol five-dimensional questionnaire; EQ-VAS, EQ-visual Analogue Scale HI; hearing impairment;

Model 1 was adjusted for age and sex. Model 2 was adjusted for age (elderly), sex, household income, education level, spouse, smoking status, alcohol intake, and regular exercise. Model 3 was adjusted for age, sex, household income, education level, spouse, smoking status, alcohol intake, regular exercise, diabetes, hypertension, hypercholesterolemia, eGFR level, and stress level. Model 4 was adjusted for age, sex, household income, education level, spouse, smoking status, alcohol intake, regular exercise, diabetes, hypertension, hypercholesterolemia, eGFR level, stress level, and tinnitus.

Unadjusted and adjusted ORs of HI for moderate or severe problems in each dimension of EQ-5D are shown in Table 3. When unadjusted for covariates, subjects with HI had a greater prevalence of problems for all EQ-5D dimensions than control group. However, after adjusting for socio-demographic factors, health-related life style behaviors, psychological stress, and comorbidities, this significance disappeared, with the exception of mobility in the mild HI group (OR, 1.398; 95% CI, 1.005–1.946, *p* = 0.047).

**Table 3:** Unadjusted and adjusted odds ratios (95% confidence intervals) of different degrees of hearing impairment for health-related quality of life.

	No HI (n = 13,802)	OR of Mild HI (n = 1,757)	*p* value	OR of Moderate to severe HI (n = 890)	*p* value
**Unadjusted**					
** Mobility**	1.0	5.863 (5.155–6.669)	< 0.001	7.011 (5.824–8.441)	< 0.001
** Self-care**	1.0	4.931 (3.984–6.104)	< 0.001	6.995 (5.436–9.000)	< 0.001
** Usual activity**	1.0	4.692 (3.999–5.503)	< 0.001	6.314 (5.135–7.763)	< 0.001
** Pain/discomfort**	1.0	2.546 (2.243–2.890)	< 0.001	2.305 (1.913–2.778)	< 0.001
** Anxiety/depression**	1.0	1.756 (1.484–2.078)	< 0.001	1.655 (1.303–2.102)	< 0.001
**Model 1**					
** Mobility**	1.0	1.533 (1.294–1.815)	< 0.001	1.368 (1.112–1.682)	0.003
** Self-care**	1.0	1.087 (0.851–1.387)	0.503	1.138 (0.863–1.502)	0.359
** Usual activity**	1.0	1.257 (1.045–1.511)	0.015	1.305 (1.037–1.642)	0.023
** Pain/discomfort**	1.0	1.311 (1.134–1.515)	< 0.001	1.030 (0.849–1.248)	0.766
** Anxiety/depression**	1.0	1.211 (0.984–1.492)	0.071	1.055 (0.803–1.387)	0.698
**Model 2**					
** Mobility**	1.0	1.479 (1.073–2.040)	0.017	1.242 (0.846–1.825)	0.268
** Self-care**	1.0	0.740 (0.463–1.182)	0.208	1.079 (0.668–1.742)	0.756
** Usual activity**	1.0	1.141 (0.793–1.643)	0.476	1.106 (0.726–1.685)	0.638
** Pain/discomfort**	1.0	1.248 (0.944–1.651)	0.119	1.043 (0.767–1.419)	0.788
** Anxiety/depression**	1.0	1.187 (0.793–1.778)	0.404	0.943 (0.574–1.548)	0.815
**Model 3**					
** Mobility**	1.0	1.462 (1.049–2.037)	0.025	1.276 (0.870–1.872)	0.211
** Self-care**	1.0	0.713 (0.436–1.166)	0.177	1.110 (0.683–1.802)	0.673
** Usual activity**	1.0	1.042 (0.720–1.509)	0.826	1.116 (0.725–1.717)	0.618
** Pain/discomfort**	1.0	1.254 (0.947–1.661)	0.114	1.088 (0.790–1.499)	0.604
** Anxiety/depression**	1.0	1.185 (0.781–1.796)	0.424	0.968 (0.557–1.684)	0.909
**Model 4**					
** Mobility**	1.0	1.398 (1.005–1.946)	0.047	1.139 (0.775–1.674)	0.508
** Self-care**	1.0	0.674 (0.410–1.108)	0.119	1.277 (0.755–2.158)	0.990
** Usual activity**	1.0	0.970 (0.671–1.403)	0.871	0.954 (0.623–1.459)	0.826
** Pain/discomfort**	1.0	1.189 (0.894–1.582)	0.235	0.970 (0.699–1.346)	0.853
** Anxiety/depression**	1.0	1.112 (0.730–1.692)	0.621	0.856 (0.498–1.473)	0.575

Abbreviations: OR, odds ratio; HI, hearing impairment

Model 1 was adjusted for age and sex. Model 2 was adjusted for age, sex, household income, education level, spouse, smoking status, alcohol intake, and regular exercise. Model 3 was adjusted for age, sex, household income, education level, spouse, smoking status, alcohol intake, regular exercise, diabetes, hypertension, hypercholesterolemia, eGFR level, and stress level. Model 4 was adjusted for age, sex, household income, education level, spouse, smoking status, alcohol intake, regular exercise, diabetes, hypertension, hypercholesterolemia, eGFR level, stress level, and tinnitus.

## Discussion

Hearing impairment is an invisible handicap and one of the most common chronic conditions of later life [23], and can impose heavy social and economic burdens on individuals, families, communities, and countries. HI tends to isolate people from friends and family members because it interferes with communication, and thus, untreated HI can have considerable social, psychological, cognitive, and health consequences [24–28]. The present study revealed a significant negative association between disabling hearing loss and EQ-VAS in a population-based sample of Korean adults. EQ-5D and EQ-VAS are preference-based generic measures of HRQoL and are used as outcome measures that encompass physical, emotional, and social dimensions in different populations. In the present study, unadjusted comparisons of HRQoL for both EQ-5D and EQ-VAS, showed impairments in subjects with HI compared with control group. After adjusting for covariates associated with the prevalence of HI and HRQoL [11, 18–20], EQ-VAS impairment in the moderate to severe HI remained significant, but EQ-5D impairment was lost. These results might be due to the different natures of the assessment tools used [1, 3]. Although EQ-5D scores may be able to detect subtle aspects that cannot be accessed by asking participants about their symptoms, its questions limit responders’ descriptions of their health states to specific dimensions. Furthermore, whereas EQ-5D measures HRQoL indirectly using five domains, EQ-VAS measures HRQoL based on participants’ subjective feelings, and thus, EQ-VAS provides a better assessment of disease severity [14, 29].

Assessments of HRQoL deterioration due to HI can be made using several instruments, which can be classified as hearing-specific HRQoL instruments or generic preference-based HRQoL instruments [30–32]. Whereas hearing-related HRQoL instruments are specifically designed to assess the effects of HI on mental and physical health and perceived benefits of hearing aids, generic HRQoL measures do not focus on any particular disorder or treatment, but rather on self-perceived overall health status [30–32]. The advantage of generic instruments is that a single instrument can be used to detect changes effectively in different aspects of patient health status and to enable comparisons across a range of conditions or diseases, and thus, it is useful for economic evaluations and healthcare decision-making as decisions can be based on a common measure and applied consistently across evaluations [33]. Furthermore, the EQ-5D and EQ-VAS have simple structures and are easily administered in clinical practice.

In a previous report [11], associations between hearing loss or tinnitus and HRQoL were evaluated using KNHANES data. It was shown hearing loss with tinnitus had a considerable impact on HRQoL, and that those with hearing loss without tinnitus had better HRQoLs than those with normal hearing with tinnitus. Since the previous study did not determine the impact of disabling HI on HRQoL, and they revealed tinnitus is important adverse effector of HRQoL as a common comorbid of HI, we included the presence of tinnitus as covariate to determine the impact of different severities HI on HRQoL in the present study.

Since HI is a common sensory impairment in the elderly, the prevalence of hearing loss will continue to rise as the global population ages [8]. The prevalence of disabling HI have been increased rapidly; World Health Organization (WHO) estimated there were 250 million people with disabling HI worldwide in 2001[4], and in 2012, approximately 360 million people (5.3% of the world's population and one-third of elderly) were reported to be affected by disabling HI [5]. In addition, the prevalence of moderate to severe hearing loss among the elderly are higher in South Asia, Asia Pacific, and Sub-Saharan Africa than elsewhere [5]. Although hearing loss is highly prevalent in Asian elderly and most can be successfully rehabilitated using appropriate hearing aids, the national prevalence of hearing aid use in South Korea is low as compared with values reported by other countries [6, 7, 34, 35]. In a Korean study, only 15.9% of participants aged 60 or older with disabling HI used a hearing aid, whereas the overall prevalence of hearing aid use among US elders aged 70 and older was reported to be 19.1% [6, 34]. The reasons for a low proportion of hearing aid usage among Korean adults with disabling HI may be multifactorial; financial issues (the cost of initial purchase and maintenance), negative beliefs about hearing aids, improper information regarding their use, or lack of a perceived hearing handicap [6, 7, 34–36]. An understanding of the effects of HI of different severities on HRQoL in Korean adults might help the acceptance of therapeutic intervention.

This study has several limitations. First, some response bias may have been introduced when subjects were asked questions about lifestyle habits and psychological stress. Second, information about HRQoL obtained using EQ-5D and EQ-VAS questionnaires were more limited than hearing-related HRQoL instruments [30–32, 37]. Third, the present study was conducted using a cross-sectional design, and thus, we cannot rule out the possibility of reverse causality, for example, HI might be a cause of impaired HRQoL, and conversely impaired HRQoL might provoke HI. Nonetheless, the results of this present study are reliable and of value, because the study was conducted using nationally representative, population-based data.

In conclusion, moderate to severe hearing loss in Korean adults was found to be associated with reduced HRQoL as determined by EQ-VAS. This finding highlights the need for improved methods to identify individuals with hearing loss at an early stage, and the understanding the HRQoL for disabling HI might be helpful to guide the hearing aids and auditory rehabilitation.
